# Highly Efficient Conversion of Motor Neuron-Like NSC-34 Cells into Functional Motor Neurons by Prostaglandin E_2_

**DOI:** 10.3390/cells9071741

**Published:** 2020-07-21

**Authors:** Hiroshi Nango, Yasuhiro Kosuge, Masaki Sato, Yoshiyuki Shibukawa, Yuri Aono, Tadashi Saigusa, Yoshihisa Ito, Kumiko Ishige

**Affiliations:** 1 Laboratory of Pharmacology, School of Pharmacy, Nihon University, 7-7-1 Narashinodai, Funabashi-shi, Chiba 274-8555, Japan; phhi16003@g.nihon-u.ac.jp (H.N.); yoshihisa.ito@yok.hamayaku.ac.jp (Y.I.); 2 Department of Physiology, Tokyo Dental College, 2-9-18 Kanda-Misakicho, Chiyoda-ku, Tokyo 101-0061, Japan; smasaki@tdc.ac.jp (M.S.); yshibuka@tdc.ac.jp (Y.S.); 3 Department of Biology Tokyo Dental College, 2-9-7 Kandasurugadai, Chiyoda-ku, Tokyo 101-0062, Japan; 4 Department of Pharmacology, School of Dentistry at Matsudo, Nihon University, 2-870-1 Sakaechonishi, Matsudo-shi, Chiba 271-8587, Japan; aono.yuri1@nihon-u.ac.jp (Y.A.); saigusa.tadashi@nihon-u.ac.jp (T.S.); 5 Pharmacy Education Center, Yokohama University of Pharmacy, 601 Matanocho, Totuka-ku, Yokohama 245-0066, Japan

**Keywords:** prostaglandin E_2_, motor neuron, neuronal differentiation, neurite outgrowth, voltage-gated sodium current, action potential, acetylcholine release

## Abstract

Motor neuron diseases are a group of progressive neurological disorders that degenerate motor neurons. The neuroblastoma × spinal cord hybrid cell line NSC-34 is widely used as an experimental model in studies of motor neuron diseases. However, the differentiation efficiency of NSC-34 cells to neurons is not always sufficient. We have found that prostaglandin E_2_ (PGE_2_) induces morphological differentiation in NSC-34 cells. The present study investigated the functional properties of PGE_2_-differentiated NSC-34 cells. Retinoic acid (RA), a widely-used agent inducing cell differentiation, facilitated neuritogenesis, which peaked on day 7, whereas PGE_2_-induced neuritogenesis took only 2 days to reach the same level. Whole-cell patch-clamp recordings showed that the current threshold of PGE_2_-treated cell action potentials was lower than that of RA-treated cells. PGE_2_ and RA increased the protein expression levels of neuronal differentiation markers, microtubule-associated protein 2c and synaptophysin, and to the same extent, motor neuron-specific markers HB9 and Islet-1. On the other hand, protein levels of choline acetyltransferase and basal release of acetylcholine in PGE_2_-treated cells were higher than in RA-treated cells. These results suggest that PGE_2_ is a rapid and efficient differentiation-inducing factor for the preparation of functionally mature motor neurons from NSC-34 cells.

## 1. Introduction

Motor neurons (MNs) reside in the central nervous system and control muscle activities [1]. There are two types of MNs: upper and lower MNs. In particular, lower MNs, located in the ventral horn of the spinal cord gray matter, form unique and irreplaceable function axons with irreplaceable functions that project directly to the endplate of peripheral muscles [2]. On receiving glutamate from upper MNs, lower MNs fire action potentials, thus releasing acetylcholine (ACh) at the neuromuscular junctions. The ACh binds to nicotinic ACh receptors on muscles causing muscle contractions. Indeed, MNs isolated from mouse spinal cords are identified as long neurite-bearing cells that express neuronal markers including neuronal cytoskeletal proteins, neurofilaments, and choline acetyltransferase (ChAT), in addition to MN-specific markers such as HB9 and Islet-1 [3]. Moreover, previous studies have reported that the mRNAs of AMPA and NMDA glutamate receptors are expressed in mouse spinal cord MNs [4] and that lumbar MNs of adult mice fire action potentials [5].

Loss of lower MN function causes progressive and fatal neurodegenerative diseases such as amyotrophic lateral sclerosis and spinal muscular atrophy. Cultured primary MNs and embryonic stem cells- or induced pluripotent stem cells-derived MNs significantly contribute to better understanding the pathogenesis of these diseases and the development of therapeutic strategies. However, these cultures have limitations, including low yields (e.g., 30,000 MNs from 5-month-old mice [3]) and limited purity (e.g., ~70% MNs from stem cells [6]). Therefore, easily accessible and stable immortal MN cell line models can overcome these limitations, such as NSC-34 cells [7], NG108-15 cells [8] and VSC4.1 cells [9]. Only a few studies have focused on the maturation properties of MNs in these cells.

NSC-34 is the most common MN cell line produced by the fusion of mouse neuroblastoma cells with MNs from the spinal cord of mouse embryos [7]. NSC-34 cells can be differentiated in culture medium containing a low concentration of serum. Differentiated NSC-34 cells exhibit the unique morphological and physiological characteristics of primary MNs (e.g., neurite outgrowth, neurofilament proteins expression, and synthesis and storage of ACh) [7,10], meaning these cells can be used as an in vitro experimental model for the study of MN dysfunction caused by various neurotoxic events such as glutamate- [11] and oxidative stress-induced cell death [12]. We have previously reported that prostaglandin E_2_ (PGE_2_), a lipid mediator produced from arachidonic acid, induced cell death in differentiated NSC-34 cells [13,14]. In contrast, a recent study has demonstrated that PGE_2_ promotes tissue regeneration in several organs such as the bone marrow, colon, and liver [15]. In the adult mouse brain, meloxicam and nimesulide, cyclooxygenases-2 inhibitors, suppressed neurogenesis [16]. Moreover, phospholipase A_2_-activating protein homozygous null mice were perinatal lethal with a decrease in brain PGE_2_ levels and less mature and differentiated neurons in the brain [17]. Previous studies have found that PGE_2_ accelerates neurite outgrowth, a morphological marker of neuronal differentiation, in NG108-15 cells [18] and ND7/23 cells [19]. We previously reported that PGE_2_ suppressed cell proliferation and induced neurite outgrowth in undifferentiated NSC-34 cells [20]. These data suggest that PGE_2_ plays an important role in neuronal differentiation and development. However, neural features of the cells differentiated by PGE_2_ still remain to be elucidated. The aim of this study is to elucidate the maturation state and functional properties of PGE_2_-differentiated NSC-34 cells.

## 2. Materials and Methods

### 2.1. Reagents

Dulbecco’s modified Eagle Medium (DMEM), choline chloride and neostigmine methyl sulfate were obtained from Sigma-Aldrich (St. Louis, MO, USA). DMEM/F12, fetal bovine serum (FBS), Penicillin-Streptomycin, MEM non-essential amino acid (NEAA), Hoechst 33,258 and propidium iodide (PI) were acquired from Thermo Fisher Scientific Inc (Waltham, MA, USA). RA, sodium hydrogen L (+)-Glutamate monohydrate (Glutamate), tetrodotoxin (TTX) and LDH-Cytotoxic Test Wako were acquired from Wako Pure Chemical Industries (Osaka, Japan). PGE_2_ was acquired from Tokyo Chemical Industry Co. Ltd. (Tokyo, Japan). PGE_2_ and RA were dissolved in ethanol (EtOH). TTX was dissolved in Milli-Q water (Millipore, Burlington, MA, USA).

### 2.2. Cell Culture

The MN-like cell line NSC-34 (provided by Dr Neil Cashman, University of Toronto, Toronto, ON, Canada) was passaged and maintained in DMEM containing 10% FBS and 1% penicillin-streptomycin in a humidified atmosphere containing 5% CO_2_ at 37 °C. Cultures underwent 5–15 passages. For the induction of neurite outgrowth, NSC-34 cells were seeded at a density of 5000 cells/cm^2^. After 24 h, different fresh media were used: (1) treatment with 30 μM PGE_2_ in DMEM containing 10% FBS and 1% penicillin-streptomycin; (2) treatment with 10 μM RA in DMEM/F12 containing 0.5% FBS, 1% penicillin-streptomycin and 1% NEAA. RA treatment was previously used for the differentiation of NSC-34 cells [21]. The medium was changed every 2–3 days.

### 2.3. Neurite Outgrowth Assay

A neurite outgrowth analysis was performed according to our previous method [20,22]. Briefly, phase-contrast micrographs were captured by inverted microscopy (IX71, Olympus, Tokyo, Japan), after which 50 cells per condition were randomly chosen for counting neurite-bearing cells. Neurite outgrowth was quantified as the percentage of cells bearing neurite processes >1 cell diameter in length.

### 2.4. Cell Viability Assay

The cell viability of NSC-34 cells was determined by an LDH assay using an LDH-Cytotoxic Test Wako, as previously described [20,23]. LDH release was assessed according to the manufacturers’ instructions. Briefly, the supernatants of the cells, treated with vehicle (EtOH) for 2 days, 30 μM PGE_2_ for 2 days, or 10 μM RA for 7 days, were collected in new plates and then mixed with the LDH substrate solution. After incubation for 45 min at room temperature, the reaction was stopped by adding 0.5 M HCl. The amount of diformazan was determined by measuring its absorbance with a microplate reader (SH-1000Lab, Corona Electric, Ibaraki, Japan) at a test wavelength of 570 nm. The LDH release of each treatment group was calculated as the percentage of LDH release from the cells treated with 0.2% Tween-20.

### 2.5. Nuclear Staining Assay

A nuclear staining assay was performed as previously described [20]. Briefly, in the incubation period, Hoechst 33,258 and PI were added to the culture medium of the cells treated with vehicle (EtOH) for 2 days, 30 μM PGE_2_ for 2 days, or 10 μM RA for 7 days (and finally, 25 μg/mL for 30 min). The images were collected with an inverted fluorescence microscope (IX71, Olympus, Tokyo, Japan). Cell mortality was quantified by expressing the number of PI-positive cells as a percentage of the number of Hoechst 33,258-positive cells. In a blind manner, at least 50 cells per condition were counted using ImageJ (Wayne Rasband National Institutes of Health, Bethesda, MD, USA).

### 2.6. Whole-Cell Patch-Clamp Recording

Whole-cell patch-clamp was performed on non-neurite-bearing cells treated with a vehicle (EtOH) for 2 days and neurite-bearing cells treated with 30 μM PGE_2_ for 2 days or with 10 μM RA for 7 days under voltage- and current-clamp conditions according to a previously method described [24]. For the recordings, the medium was changed to artificial cerebrospinal fluid (aCSF) containing 136 mM NaCl, 5 mM KCl, 2.5 mM CaCl_2_, 0.5 mM MgCl_2_, 10 mM HEPES, 10 mM glucose, and 12 mM NaHCO_3_, pH 7.4 by Tris. A Na^+^-free aCSF was prepared by substituting 136 mM NaCl in the standard aCSF with an equimolar concentration of tetraethylammonium chloride. Patch pipettes (3–5 MΩ) were made from capillary tubes by a DMZ-Universal Puller (Zeitz-Instruments, Martinsried, Germany) and then filled with an intracellular solution (150 mM KCl, 10 mM HEPES and 2 mM Mg-ATP pH 7.2 by Tris). Voltage- and current-clamp recordings were carried out using a L/M-EPC-7+ patch-clamp amplifier (Heka Elektronik, Lambrecht, Germany) and an Axopatch-1D patch-clamp amplifier (Axon Instruments, Foster City, CA, USA), respectively. All recordings were digitized at 10 kHz with a DigiData 1440A analog-to-digital interface (Molecular Device, San Jose, CA, USA) and stored using pCLAMP (Molecular Device, San Jose, CA, USA). The action potential amplitude was measured from the threshold potential to the peak potential. Inward current amplitudes were normalized to the single cell capacitance values and were expressed as current densities (pA/pF). All experiments were performed at 30 °C.

### 2.7. Western Blotting

The Western blot analysis was performed as described previously [20,22]. Briefly, protein extracts from the cells treated with vehicle (EtOH) for 2 days, 30 μM PGE_2_ for 2 days, or 10 μM RA for 7 days were separated on SDS-polyacrylamide gels and transferred to polyvinylidene difluoride membranes (Millipore, Billerica, MA, USA) using a Transblot SD Semi-Dry Transfer Cell (BioRad, Hercules, CA, USA). The membranes were treated with anti-MAP2 monoclonal antibody (#8707, 1:1000; Cell Signaling Technology, Danvers, MA, USA), anti-Syanaptophysin antibody (#5461, 1:1000; Cell Signaling Technology, Danvers, MA, USA), anti-HB9 antibody (sc-515769, 1:500; Santa Cruz Biotechnology, Dallas, TX, USA), anti-Islet-1 antibody (ab109517, 1:1000, abcam, Cambrige, MA, USA), anti-ChAT antibody (AB144P, 1:500; Millipore, Burlington, MA, USA), and anti-βactin antibody (A5441, 1:2000, Sigma-Aldrich, St. Louis, MO, USA) overnight at 4 °C. Immunoreactive bands were detected by an ECL detection system (GE Healthcare Life Sciences, St Chalfont, Giles, USA) after incubation with a horseradish peroxidase-conjugated secondary antibody (1:10,000–20,000; Santa Cruz Biotechnology, Dallas, TX, USA) for 1 h at room temperature. The optical density of the bands was quantified using Scion imaging software (Scion, Frederick, MD, USA).

### 2.8. Acetylcholine Release and Quantification

The quantification of ACh release was carried out as described previously [25,26]. The cells treated with vehicle (EtOH) for 2 days, 30 μM PGE_2_ for 2 days, or 10 μM RA for 7 days were incubated with standard aCSF containing 10 μM choline and 100 nM neostigmine (500 μL) for 60 min at 37 °C and 5% CO_2_. In order to determine ACh release, this buffer (250 μL) was removed and replaced with an equal volume of fresh buffer. After 10 min, a 100 μL aliquot of this buffer was collected and centrifuged. The supernatants were immediately frozen in liquid nitrogen and stored at −80 °C. The supernatants were mixed with an internal standard of isopropyl homocholine (IPHC) and injected into a HPLC system (HTEC-500; Eicom, Kyoto, Japan). ACh was separated on an Eicompak AC-GEL column (particle size 4 μm, 2.0 × 150 mm; Eicom, Kyoto, Japan) using a carbonate buffer as the mobile phase at a flow rate of 150 μL/min. A post-column enzyme reactor (AC-ENZYM II; Eicom, Kyoto, Japan) containing acetylcholinesterase and choline oxidase was used to produce hydrogen peroxide from ACh. The hydrogen peroxide levels were electrochemically detected using a platinum electrode at +450 mV against an Ag/AgCl reference electrode (WE- 3G; Eicom, Kyoto, Japan). The separation column and post-column enzyme reactor were maintained at 33 °C. The amount of ACh in the supernatant was calculated by comparing it to the area values of standard ACh and IPHC solutions, after which the data were normalized to cell numbers counted by hemocytometry.

### 2.9. Statistical Analysis

All data are expressed as mean ± standard error of the mean (SEM) or standard deviation (SD). Statistical significance was assessed using a one-way or two-way analysis of variance (ANOVA) followed by a post hoc Tukey’s multiple test or a Student’s t-test. A *p* < 0.05 was considered reflective of statistical significance.

## 3. Results

### 3.1. Effect of PGE_2_ and RA on Neurite Outgrowth

**Figure 1:**
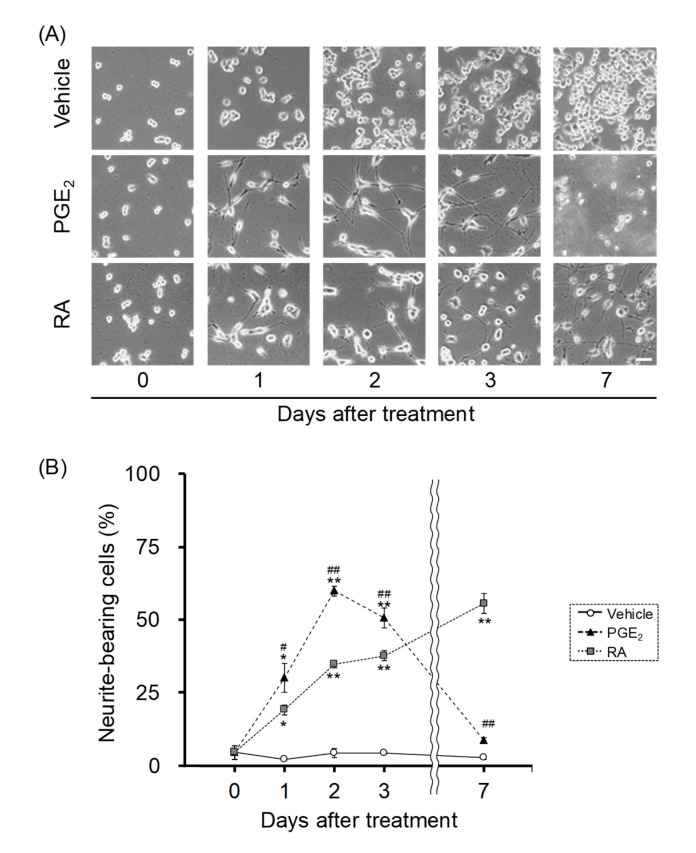
The effects of prostaglandin E_2_ (PGE_2_) and retinoic acid (RA) on neurite outgrowth. Undifferentiated NSC-34 cells were treated with a vehicle (EtOH), 30 μM PGE_2_ or 10 μM RA. (**A**) Photographs show typical phase-contrasts in each treatment group. Scale bar indicates 50 μm. (**B**) Graph shows the quantitative analysis of cells bearing neurite, expressed as the percentage of cells bearing neurites. Each value represents the mean ± SEM (*n* = 4). * *p* < 0.05, ** *p* < 0.01 vs. vehicle-treated cells at same day. ^#^ *p* < 0.05, ^##^ *p* < 0.01 vs. RA-treated cells at same day.

RA is a potent and widely-used signaling factor that stimulates the differentiation of embryonic stem cells and stem/progenitor cells in vitro [27,28]. We compared the effects of PGE_2_ and RA on neurite outgrowth in undifferentiated NSC-34 cells, and evaluated the time-dependency of PGE_2_- and RA-induced neurite outgrowth by phase-contract microscopy. Neurite-bearing cells were observed in cells treated with PGE_2_ (30 μM) and RA (10 μM) from treatment day 1 onwards (Figure 1A). Treatment with PGE_2_ for 7 days led to drastic cell death and the subsequent detachment of the adherent cells from the bottom of the plate (Figure 1A). In contrast, vehicle-treated cells were still round in shape after 7 days (Figure 1A). Exposure of undifferentiated NSC-34 cells to PGE_2_ (30 μM) increased the percentage of neurite-bearing cells that reached a peak at day 2 (59.8 ± 1.8%) (Figure 1B). Treatment with RA (10 μM), which was commonly used concentration for the differentiation of NSC-34 cells [21], increased the percentage of neurite-bearing cells in a time-dependent manner (Figure 1B). The percentage of neurite-bearing cells treated with RA remained at 34.6 ± 1.1% on day 2. On day 7, it reached the same level as in the treatment with PGE_2_ on day 2 (55.5 ± 3.5%) (Figure 1B). The percentage of neurite-bearing cells treated with PGE_2_ was significantly higher than in those treated with RA on days 1 to 3 (Figure 1B).

**Figure 2:**
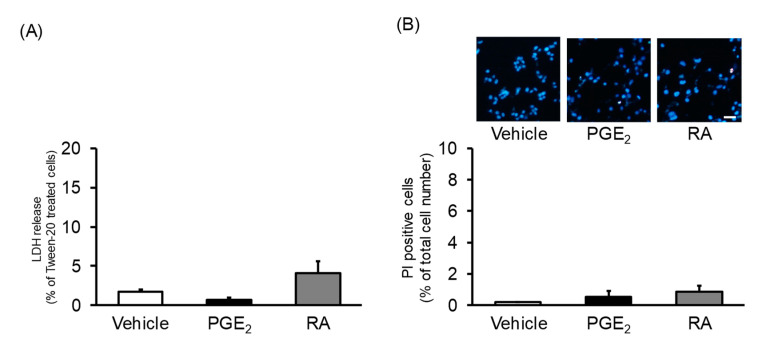
The effects of PGE_2_ and RA on viability. Undifferentiated NSC-34 cells were treated with a vehicle (EtOH), 30 μM PGE_2_ for 2 days, or 10 μM RA for 7 days. (**A**) Graph shows the percentage of released LDH of PGE_2_- and RA-treated cells relative to that of Tween-20-treated cells. Each value represents the mean ± SEM (*n* = 4). (**B**) Photographs show representative fluorescence microscopy images of typical Hoechst 33,258/propidium iodide (PI) double staining in each treatment group. Scale bar indicates 50 μm. Graph shows quantitative analysis of PI-positive cells, expressed as the ratio of PI-positive cells to Hoechst 33,258-positive cells. Each value represents the mean ± SEM (*n* = 4).

Next, we investigated the cytotoxicity of PGE_2_ and RA in undifferentiated NSC-34 cells using an LDH release assay (Figure 2A) and Hoechst 33,258/PI double staining (Figure 2B) using the same time schedule as shown in Figure 1. LDH release and the percentage of PI-positive cells in the vehicle-treated cells were 0.2 ± 0.1% and 1.8 ± 0.2%, respectively. Exposure to PGE_2_ for 2 days did not increase LDH release (0.7 ± 0.3%) or PI-positive cells (0.6 ± 0.3%). Likewise, a treatment with RA for 7 days did not affect the level of LDH release (5.1 ± 0.8%) or the ratio of PI-positive cells (0.9 ± 0.4%).

### 3.2. Action Potential Generation in Differentiated NSC-34 cells

**Figure 3:**
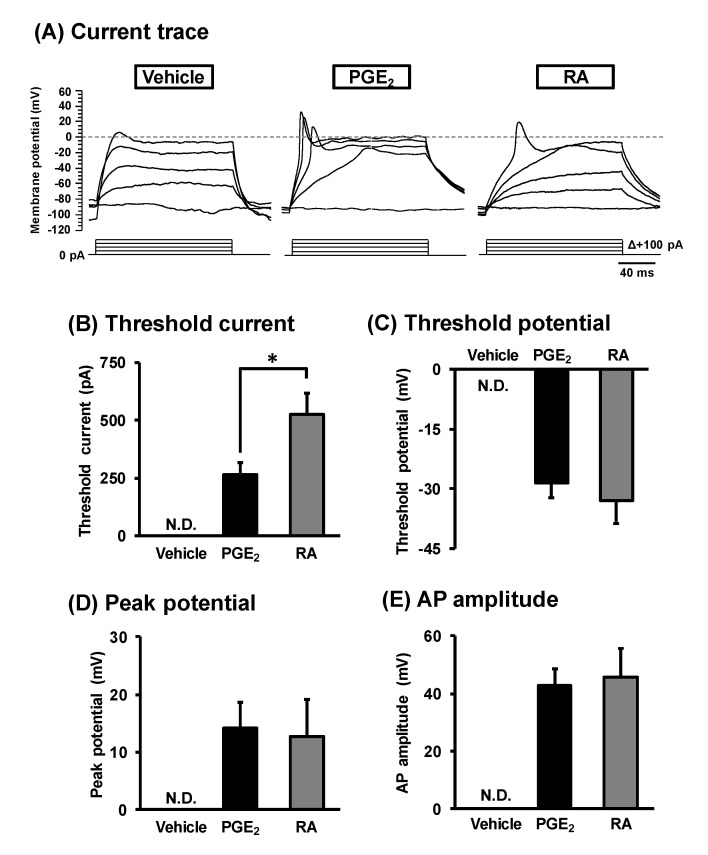
Generation of an action potential (AP) in NSC-34 cells. Undifferentiated NSC-34 cells were treated with a vehicle (EtOH), 30 μM PGE_2_ for 2 days, or 10 μM RA for 7 days. (**A**) Representative traces of an action potential recorded by depolarization current steps from 0 to +400 pA (150 ms in duration) with 100 pA increments in each treatment group. Graphs show threshold current (**B**), threshold potential (**C**), AP peak potential (**D**), and AP amplitude (**E**) in each treatment group. Each value represents the mean ± SD (PGE_2_; *n* = 16, RA; *n* = 12). * *p* < 0.05. N.D. means not detected.

The generation of action potentials is an important physiological property of mature neurons [29,30]. To assess whether action potentials are generated in NSC-34 cells, we performed current-clamp recordings on non-neurite-bearing cells treated with a vehicle for 2 days and neurite-bearing cells treated with PGE_2_ (30 μM) for 2 days or RA (10 μM) for 7 days. As shown in Figure 3A, action potential was observed in response to 150 ms of depolarization current pulses from 0 pA to +400 pA with 100 pA increments in the cells treated with PGE_2_ and RA. In contrast, vehicle-treated cells did not generate an action potential. The current threshold for action potential generation of PGE_2_-treated cells (262.5 ± 55.4 pA) was significantly lower than that of RA-treated cells (525.0 ± 92.4 pA) (Figure 3B). Threshold potential (Figure 3C), peak potential (Figure 3D) and amplitude of the first spike (Figure 3E) in PGE_2_-treated cells were the same as those of RA-treated cells.

### 3.3. Characterization of Voltage-Dependent Inward Currents

**Figure 4:**
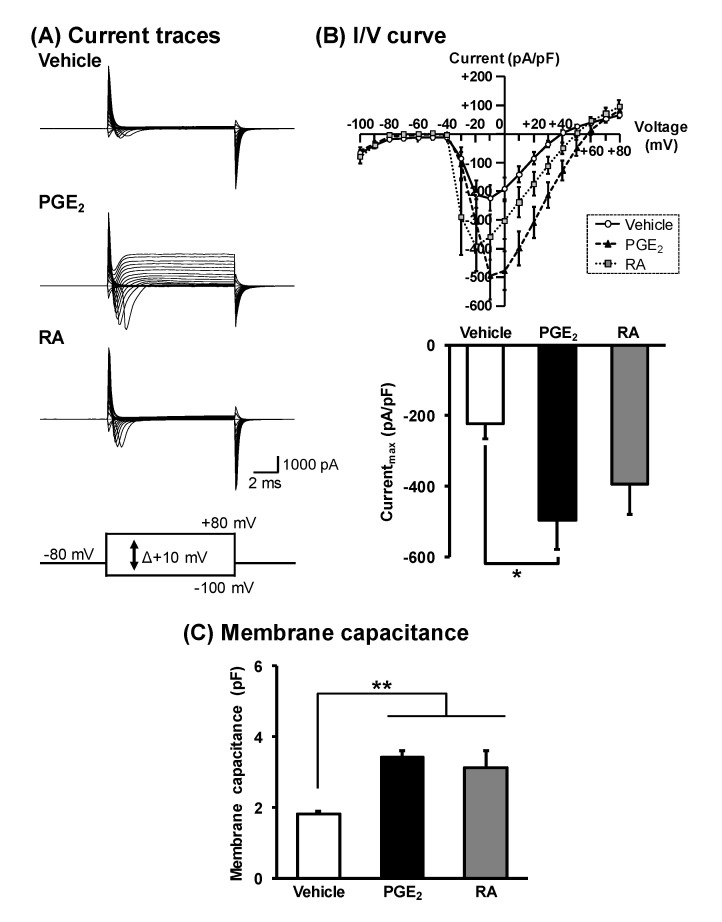
Generation of inward currents in NSC-34 cells. Undifferentiated NSC-34 cells were treated with a vehicle (EtOH), 30 μM PGE_2_ for 2 days, or 10 μM RA for 7 days. (**A**) Representative inward current traces recorded by depolarization voltage pulses from –100 mV to +80 mV in 10 mV steps from a holding potential of –80 mV in each treatment group. (**B**) Upper graph shows current–voltage relationships recorded by voltage pulses in each treatment group. Lower graph shows the peak current densities of each treatment group. Each value represents the mean ± SD (*n* = 18). * *p* < 0.05. (**C**) Graph shows membrane electrical capacitance in each treatment group. Each value represents the mean ± SD (*n* = 18). ** *p* < 0.01.

Voltage-gated Na^+^ channels play the most important role in the generation of action potential [31,32]. We examined whether voltage-dependent Na^+^ channels are functionally expressed in NSC-34 cells using voltage-clamp recordings. Voltage-dependent inward currents were observed in response to depolarizing voltage steps ranging from −100 to +80 mV with a holding potential of -80 mV in each treatment (Figure 4A). The peak current density of PGE_2_-differentiated cells (494.8 ± 84.2 pA/pF) was significantly larger than that of vehicle-treated cells (224.0 ± 42.9 pA/pF). However, the peak current density of RA-differentiated cells (393.6 ± 85.4 pA/pF) tended to be increased compared to that of vehicle-treated cells, although no significant difference was observed between the two (Figure 4B). Individual inward currents were normalized to the membrane capacitance of each cell (Figure 4C).

**Figure 5:**
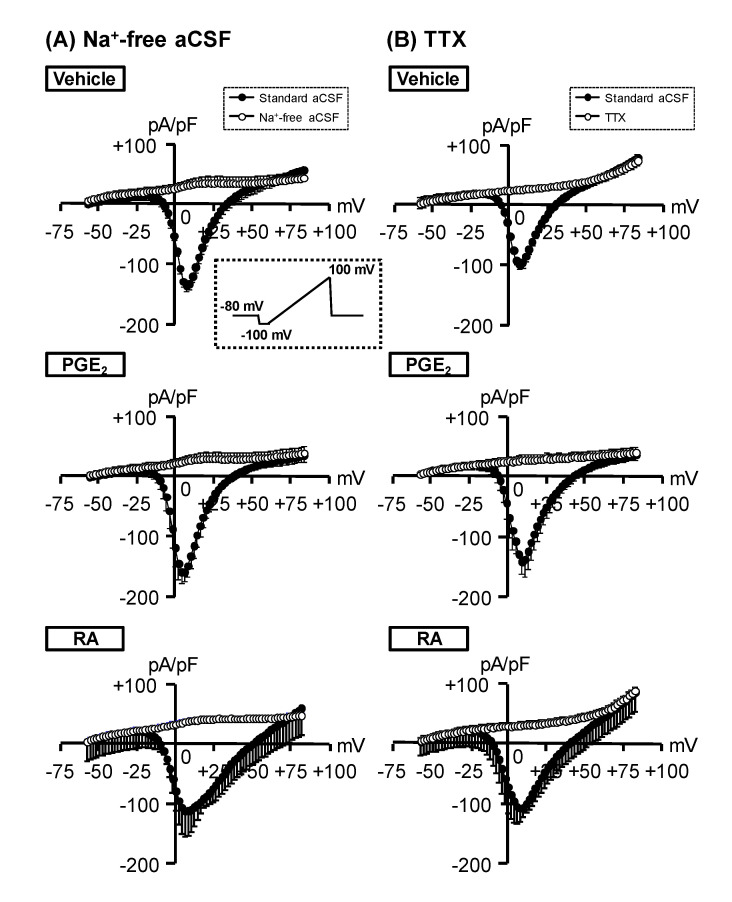
The effect of Na^+^-free artificial cerebrospinal fluid (aCSF) or tetrodotoxin (TTX) perfusion on voltage-dependent inward current. Undifferentiated NSC-34 cells were treated with a vehicle (EtOH), 30 μM PGE_2_ for 2 days, or 10 μM RA for 7 days. Ramp currents were recorded by voltage pulses of 100 ms from –100 mV to +100 mV (insert panel). Baseline was subtracted by fitting the linear portion between −100 mV and −60 mV to zero. Graphs show the current–voltage relationship of the ramp currents with or without Na^+^-free aCSF (**A**) and 1 μM TTX (**B**) in each treatment group. Each value represents the mean ± SD (*n* = 18).

Next, we assessed whether voltage-gated Na^+^ channels contributed to the activation of voltage-dependent inward currents in vehicle-, PGE_2_- and RA-treated NSC-34 cells. As shown in Figure 5A, Na^+^-free aCSF abolished the inward current in all treatment groups. Likewise, the classical Na^+^ channel blocker TTX (1 μM) completely suppressed inward currents in all treatment groups (Figure 5B).

### 3.4. Expression of Neuronal Markers in PGE_2_- and RA-Differentiated cells

**Figure 6:**
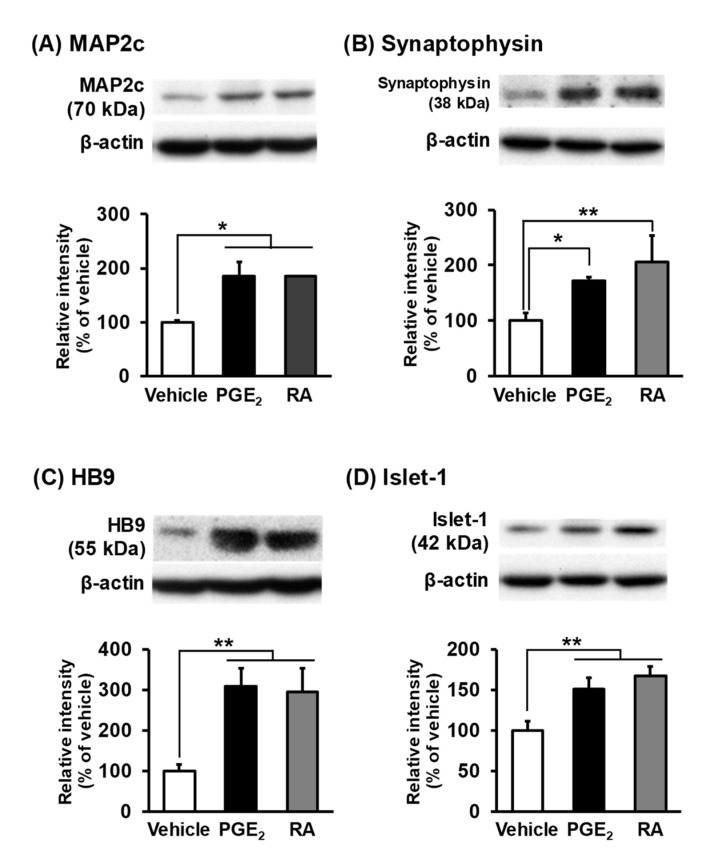
Expression of motor neuron-specific markers in NSC-34 cells. Undifferentiated NSC-34 cells were treated with a vehicle (EtOH), 30 μM PGE_2_ for 2 days, or 10 μM RA for 7 days. Photograph shows a representative result of a Western blot of low-molecular-weight microtubule-associated protein-2 (MAP2c) (**A**), synaptophysin (**B**), HB9 (**C**) and Islet-1 (**D**) with β-actin as an internal marker. Graphs show the relative densities of bands on the blots estimated quantitatively using Scion imaging software. Each value represents the mean ± SD (*n* = 3). * *p* < 0.05, ** *p* < 0.01.

We then examined the expression of neural differentiation markers such as low-molecular-weight microtubule-associated protein-2 (MAP2c), which increases in expression from the early stage of neuronal differentiation, and synaptophysin, which increases in expression from the maturation stage, through Western blotting. The MAP2c protein levels, calculated assuming a 100% intensity of MAP2c expression in vehicle-treated cells, were significantly upregulated in PGE_2_-treated cells (184.9 ± 26.5%) and RA-treated cells (185.2 ± 0.9%) (Figure 6A). Likewise, the expression of synaptophysin was significantly increased in PGE_2_-treated cells (171.9 ± 6.1%) and RA-treated cells (206.7 ± 46.7%) (Figure 6B). Moreover, we tested the expression of the MN-specific markers, the transcription factors HB9 and Islet-1, involved in the selective differentiation and maturation of MNs. HB9 protein levels were significantly increased in PGE_2_-treated cells (338.7 ± 39.6%) and RA-treated cells (328.4 ± 73.6%) (Figure 6C). The protein levels of Islet-1 were also significantly upregulated in PGE_2_-treated cells (140.5 ± 8.3%) and RA-treated cells (164.9 ± 14.6%) (Figure 6D). There were no significant differences in the expression levels of these proteins between PGE_2_-differentiated and RA-differentiated cells (Figure 6A–D)

### 3.5. Synthesis and Release of ACh in NSC-34 Cells

**Figure 7:**
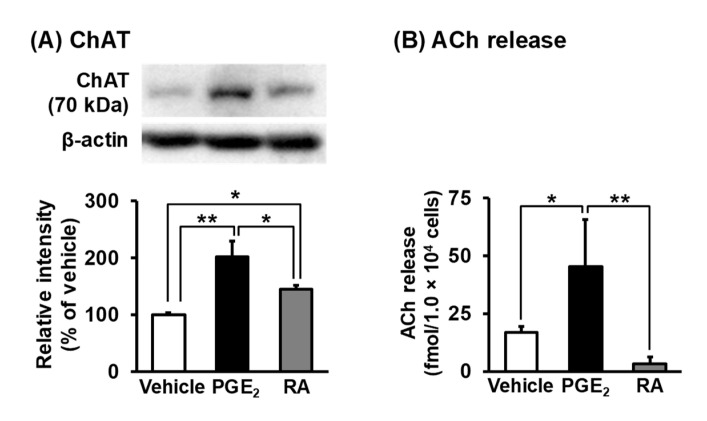
Synthesis and release of acetylcholine (ACh) in NSC-34 cells. Undifferentiated NSC-34 cells were treated with a vehicle (EtOH), 30 μM PGE_2_ for 2 days, or 10 μM RA for 7 days. (**A**) The photograph shows a representative result of the Western blotting of Choline acetyltransferase (ChAT) with β-actin as an internal marker. The open arrowhead indicates a nonspecific band recognized by the antibody. Graph shows the relative densities of bands on the blots quantitatively estimated by Scion imaging software. Each value represents the mean ± SD (*n* = 3). * *p* < 0.05, ** *p* < 0.01. (**B**) Graph shows the ACh concentration normalized per 1.0 × 10^4^ cells in each treatment group. Each value represents the mean ± SD (*n* = 4). * *p* < 0.05, ** *p* < 0.01.

To assess neuronal function after differentiation with PGE_2_ and RA, we evaluated the synthesis of ACh, a MN neurotransmitter, in NSC-34 cells. Western blotting showed that the expression of ChAT, an enzyme responsible for the synthesis of ACh, was significantly increased in PGE_2_-treated cells (202.7 ± 26.4%) and RA-treated cells (145.6 ± 6.1%) as compared to vehicle-treated cells (Figure 7A). The protein level of ChAT in PGE_2_-treated cells was significantly higher than that in RA-treated cells. Finally, we determined whether the basal release of ACh from NSC-34 cells was subject to modulation by PGE_2_ or RA. As shown in Figure 7B, the levels of ACh release in PGE_2_-treated cells (45.4 ± 20.4 fmol/1.0 × 10^4^ cells) were significantly higher than those released in vehicle-treated cells (16.8 ± 2.7 fmol/1.0 × 10^4^ cells). On the other hand, there were no significant differences in the levels of ACh release between RA-treated cells (3.13 ± 3.0 fmol/1.0 × 10^4^ cells) and vehicle-treated cells (Figure 7B).

## 4. Discussion

Neuritogenesis is an early event in neuronal differentiation characterized by the extension of neurites that will develop into axons and dendrites. The axon projects to an appropriate target and then constructs and maintains a functional synapse. MNs extend long axons projecting from the ventral horn of the spinal cord gray matter directly to peripheral muscles [2]. When an action potential reaches a presynaptic nerve terminal, ACh, a neurotransmitter, is released from MNs into the synaptic cleft and stimulates muscle fibers, causing muscle contraction [2]. It is reported that RA activates transcription factors for ventral neural patterning and MN specification [33] and is commonly used to differentiate stem cells into MNs in vitro [6,34]. RA has also been reported to inhibit proliferation, promote neurite outgrowth, and differentiate NSC-34 cells into MN-like cells [10,21,35]. Previously, we reported that PGE_2_ promoted neurite outgrowth and suppressed cell proliferation in undifferentiated NSC-34 cells [20]. However, the differentiation and maturation characteristics of MN–like NSC-34 cells induced by PGE_2_ remain unclear. In this study, we compared the morphological, electrophysiological, and biochemical properties of differentiated cells by PGE_2_- with those by RA, after which the neuronal properties of PGE_2_-treated NSC-34 cells were discussed.

Consistent with previous reports [10,21,35], the present study demonstrates that RA induces time-dependent neurite outgrowth in undifferentiated NSC-34 cells up to day 7. PGE_2_ also induced neurite outgrowth, reaching a peak level on day 2, and the percentage of neurite-bearing cells in PGE_2_-treated cells was higher than that in RA-treated-treated cells on the same day. On day 7 in RA-treated cells, it reached nearly the same level as in response to the treatment with PGE_2_ on day 2. Exposure to PGE_2_ for 2 days and RA for 7 days did not affect the viability of these cells. Nevertheless, the treatment with PGE_2_ for 7 days resulted in a drastic decrease in cell viability. Our laboratory has previously reported that PGE_2_ induced cell death in differentiated NSC-34 cells [13,14,23]. Therefore, the PGE_2_-induced cell death observed in this study was considered to be consistent with these results. These results indicate that PGE_2_ induces morphological differentiation more rapidly than RA.

The generation of action potentials is a physiological property that is important for mature neurons [36]. A previous study presented by other researchers has reported that RA-differentiated NSC-34 cells generate action potentials such as primary cultures of mouse spinal MNs [37]. Consistent with this report, this study showed the generation of action potential in NSC-34 cells differentiated by RA. We demonstrated that neurite-bearing cells differentiated by PGE_2_ also generated action potentials. Although there were no differences in the threshold potential, peak potential, or amplitude between PGE_2_- and RA-differentiated cells, the threshold currents were significantly lower in PGE_2_-differentiated cells than in RA-differentiated cells. A previous study has presumed that MNs in adult mice (postnatal age 42 days and above) lumbar spinal cord slices have smaller rheobase values than MNs at an early postnatal stage (until 11 days of giving birth) [5], suggesting that the decrease in threshold current relates to the neurological maturation in MNs in the spinal cord. In this study, we showed that the current stimulation for generating action potentials in PGE_2_-differentiated cells was significantly lower than that in RA-differentiated cells. These results suggest that PGE_2_ induces the differentiation of NSC-34 cells into electrophysiologically functional neuronal cells on day 2 and that the maturation level of these cells is higher than that of RA-differentiated cells on day 7.

It is well known that voltage-gated Na^+^ channels play the most important role in the generation of action potentials [31,32]. It has been reported that human-induced pluripotent stem cells-derived neurons show a maturation-dependent pattern of voltage-gated Na^+^ current expression and graded action potentials [38]. In addition, the action potential in MNs derived from human embryonic stem cells was completely blocked by TTX [6,39]. Taken together, these data suggest that the increase in voltage-gated Na^+^ current is essential for the initiation of an action potential. In the present study, we demonstrated that the voltage-gated inward current density in PGE_2_-differentiated cells was significantly higher than that in undifferentiated cells, whereas the density in RA-differentiated cells was comparable to that in undifferentiated cells. The inward current in each group was completely inhibited by Na^+^-free aCSF and by 1 μM TTX, suggesting that the inward current in these cells is mainly due to TTX-sensitive Na^+^ channels. These results suggest that the electro-excitability in NSC-34 cells differentiated by PGE_2_ and RA is caused by the increase in the density of voltage-dependent Na^+^ currents. It has been reported that multiple voltage-gated Na^+^ channels subtypes exist, which vary in terms of their physiological and pharmacological properties [31]. Further studies are required to identify the voltage-gated Na^+^ channels subtype(s) responsible for the excitability of MN-like NSC-34 cells promoted by PGE_2_.

Previous studies have reported that differentiated NSC-34 cells were characterized by the expression of neuronal marker proteins [7,10,21]. MAP2c, which increases in expression from early stages of neuronal differentiation, plays an essential role in neurite outgrowth and microtubule stabilization [40,41]. Synaptophysin, which increases in expression from the maturation stage, regulates neurotransmitter release [42,43]. These proteins are used as neuronal differentiation markers in NSC-34 cells differentiated by RA [10]. Consistent with previous results, we observed that the expression of these proteins increased significantly in RA-differentiated cells as compared to undifferentiated cells. Moreover, we observed that the protein levels of MAP2c and synaptophysin were up-regulated in PGE_2_-differentiated cells as well as in RA-differentiated cells. These results suggest that NSC-34 cells treated by PGE_2_ differentiated into neuron-like cells, as with the cells treated by RA. We also assessed whether the treatment of NSC-34 cells with PGE_2_ encouraged cells to develop general MN characteristics. HB9 and Islet-1 are homeodomain transcription factors that play essential roles in the selective differentiation into MNs and the formation of mature MNs [44,45]. These factors are thus used as MN-specific markers in induced pluripotent stem cells-derived MNs [6]. A previous study reported that HB9 and Islet-1 are expressed in RA-differentiated NSC-34 cells [21]. The current study found that the expression levels of HB9 and Islet-1 in PGE_2_-differentiated cells were increased as compared to those in RA-differentiated cells. These results indicate that the treatment of NSC-34 cells with PGE_2_ as well as RA promoted their differentiation to MN-like cells.

The release of ACh from cells is key to specifying the pan-neuronal properties of developing MNs [2]. Although previous studies have reported that RA-differentiated NSC-34 cells expressed ChAT [12,21], which is involved in the synthesis of ACh, in a manner that is dependent on the stage of MN maturation, the expression of this enzyme in PGE_2_-differentiated NSC-34 cells has not yet been clarified. We demonstrated that the expression of ChAT was increased in PGE_2_- and RA-differentiated cells and that the protein levels of ChAT in PGE_2_-differentiated cells were higher than those in RA-differentiated cells. We also demonstrated that undifferentiated NSC-34 cells have the capacity to release ACh and that the release of ACh from PGE_2_-differentiated NSC-34 cells was significantly higher than that from undifferentiated cells. Surprisingly, the level of ACh release from RA-differentiated cells tends to be lower than that from undifferentiated cells. It has been reported that mRNA levels of the vesicular ACh transporter in RA-differentiated NSC-34 cells remain unchanged compared with those of undifferentiated cells until 8 days after treatment [10]. Therefore, one possible explanation for the difference between the levels of ACh release from PGE_2_- and RA-differentiated cells is attributable to the differential level of expression and/or the intrinsic activity of vesicular ACh transporters. Although the mechanism underlying the difference in the levels of ACh release warrants further investigation, our findings suggest that PGE_2_ differentiates NSC-34 cells into functionally mature MNs with the ability to release ACh.

In conclusion, we demonstrated that PGE_2_ promoted the efficient conversion of NSC-34 cells into mature MN-like cells with neurite outgrowth, the capacity to generate action potentials, increased expression of MN-specific proteins, and a higher level of ACh release. A previous study has reported that RA-induced differentiated NSC-34 cells are unsuitable as experimental models for glutamate excitotoxicity in MN diseases [4]. Our findings suggest that PGE_2_ is a new promotor for the preparation of functionally mature MN-like cells. Additionally, NSC-34 cells differentiated by PGE_2_, as well as cells differentiated by RA, may be a suitable model to investigate the pathogenesis of MN diseases.
